# Hypoxia and transforming growth factor β1 regulation of long non‐coding RNA transcriptomes in human pulmonary fibroblasts

**DOI:** 10.14814/phy2.14343

**Published:** 2020-01-10

**Authors:** Lakmini K. Senavirathna, Chaoqun Huang, Samuel Pushparaj, Dao Xu, Lin Liu

**Affiliations:** ^1^ Oklahoma Center for Respiratory and Infectious Diseases Oklahoma State University Stillwater OK USA; ^2^ Lundberg‐Kienlen Lung Biology and Toxicology Laboratory Department of Physiological Sciences Oklahoma State University Stillwater OK USA

**Keywords:** hypoxia, lncRNA, TGFβ, transcriptome

## Abstract

One of the key characteristics of idiopathic pulmonary fibrosis (IPF) is accumulation of excess fibrous tissue in the lung, which leads to hypoxic conditions. Transforming growth factor (TGF) β is a major mediator that promotes the differentiation of fibroblasts to myofibroblasts. However, how hypoxia and TGFβ together contribute the pathogenesis of IPF is poorly understood. Long non‐coding RNAs (lncRNAs) have regulatory effects on certain genes and are involved in many diseases. In this study, we determined the effects of hypoxia and/or TGFβ on mRNA and lncRNA transcriptomes in pulmonary fibroblasts. Hypoxia and TGFβ1 synergistically increased myofibroblast marker expression. RNA sequencing revealed that hypoxia and TGFβ1 treatment resulted in significant changes in 669 lncRNAs and 2,676 mRNAs compared to 150 lncRNAs and 858 mRNAs in TGFβ1 alone group and 222 lncRNAs and 785 mRNAs in hypoxia alone group. TGFβ1 induced the protein expression of HIF‐1α, but not HIF‐2α. On the other hand, hypoxia enhanced the TGFβ1‐induced phosphorylation of Smad3, suggesting a cross‐talk between these two signaling pathways. In all, 10 selected lncRNAs (five‐up and five‐down) in RNA sequencing data were validated using real‐time PCR. Two lncRNAs were primarily located in cytoplasm, three in nuclei and five in both nuclei and cytoplasm. The silencing of HIF‐1α and Smad3, but not Smad2 and HIF‐2α rescued the downregulation of FENDRR by hypoxia and TGFβ1. In conclusion, hypoxia and TGFβ1 synergistically regulate mRNAs and lncRNAs involved in several cellular processes, which may contribute to the pathogenesis of IPF.

## Graphical abstract

One of the key characteristics of Idiopathic pulmonary fibrosis (IPF) is accumulation of excess fibrous tissue in the lung, which leads to hypoxic conditions. Transforming growth factor (TGF) β is a major mediator that promotes the differentiation of fibroblasts to myofibroblasts. Hypoxia and TGFβ1 synergistically regulate mRNAs and lncRNAs involved in several cellular processes, which may contribute to the pathogenesis of IPF.

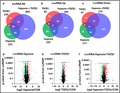

## 1 INTRODUCTION

The International Human Genome Sequencing Consortium has revealed that only <2% of human genome is encoded for proteins (Elgar & Vavouri, 2008; Wilusz, Sunwoo, & Spector, 2009). However, a majority of human genome is transcribed into non‐coding RNAs that do not have protein‐coding potential (Beermann, Piccoli, Viereck, & Thum, 2016). Based on their sizes, non‐coding RNAs are categorized into small non‐coding RNAs (20–30 nucleotides [nt]) and long non‐coding RNAs (lncRNAs) (>200 nt). Small non‐coding RNAs are further divided into several groups based on their origin and functions, including small nucleolar RNAs (snoRNAs), microRNAs (miRNAs), small interfering RNAs (siRNAs), and PIWI interacting RNA (piRNAs) (Beermann et al., 2016; Hüttenhofer, Schattner, & Polacek, 2005). snoRNAs are involved in gene regulation and chemical modification of other RNAs. miRNAs function as post‐transcriptional regulation of genes. siRNAs interfere with gene expression by degrading mRNAs. piRNAs bind PIWI proteins and represses the transposons (Beermann et al., 2016).

Nucleotide sequences of lncRNAs are poorly conserved among species. However, they may form the same three‐dimensional structures and thus perform the similar functions (Beermann et al., 2016). Unlike miRNA and other small non‐coding RNAs, the functions of lncRNAs are not well studied. Recent genome‐wide analysis conducted on lncRNAs offers an opportunity to uncover their functions at cellular levels (Baker, 2011; Liu et al., 2018; Ren, Fan, Liu, Wang, & Zhao, 2018; Song et al., 2014). Molecular mechanisms of lncRNA action vary from epigenetic regulation, RNA decoy, RNA scaffolds, and guide for proteins to cis‐ and trans‐regulators (Mercer & Mattick, 2013). LncRNAs are involved in the pathogenesis of many diseases including pulmonary fibrosis, acute respiratory distress syndrome, infectious diseases, and cancer (Huang, Yang, & Liu, 2015; Huarte, 2015; Imam, Bano, Patel, Holla, & Jameel, 2015; More et al., 2019; Winterling et al., 2014; Zhou, Wang, Shao, & Wang, 2016).

Idiopathic pulmonary fibrosis (IPF) is a fibrotic lung disease, belonging to idiopathic interstitial pneumonia. The term “idiopathic” is given for its unknown etiology, but it has several risk factors associated with it (King, Pardo, & Selman, 2011; Lee, Mira‐Avendano, Ryu, & Daniels, 2014). Cigarette smoke, genetics, and exposure to industrial dusts are main risk factors associated with IPF. Apart from them, some comorbid conditions such as obesity, diabetes, coronary heart disease, pulmonary hypertension, emphysema, and gastroesophageal reflux also contribute to the development of this disease. The main pathological feature of IPF is accumulation of extracellular matrix proteins, especially collagens, which cause the scarring of lung tissues. IPF is believed to be an epithelium‐driven disease. Repeated epithelial micro‐injuries result in the abnormal activation of lung epithelial cells, which secrete growth factors to activate many cellular pathways in fibroblasts (King et al., 2011). Transforming growth factor beta (TGFβ) is the key factor to activate fibroblasts to secrete excessive amount of extracellular matrix proteins (King et al., 2011; Leask & Abraham, 2004; Lee et al., 2014). Hypoxia is another feature in IPF (Kawakami, Mimura, Shoji, Tanaka, & Nangaku, 2014; Tzouvelekis et al., 2007). We and others have shown that hypoxia stimulates lung fibroblast proliferation (Bodempudi et al., 2014; Mizuno et al., 2009; Senavirathna et al., 2018). There is evidence that hypoxia and TGFβ signaling cross‐talk to promote fibrosis (Abdul‐Hafez, Shu, & Uhal, 2009; Qian et al., 2015; Ueno et al., 2011).

To understand the roles of lncRNAs in IPF, we performed RNA sequencing analysis to reveal genome‐wide changes of lncRNAs in hypoxia and/or TGFβ1‐treated human lung fibroblasts and further investigated the regulatory mechanisms of one of the identified lncRNAs, FENDRR by hypoxia and TGFβ1.

## 2 MATERIALS AND METHODS

### 2.1 Cell culture

Primary human pulmonary fibroblasts (HPFs) isolated from a 74‐year‐old‐male Caucasian person were purchased from PromoCell (Heidelberg, Germany, Cat. No: C‐12361). HPFs were cultured in fibroblast medium (PromoCell, Cat. No: C‐23220) with its supplements (PromoCell, Cat. No: C‐39320) containing fetal calf serum (0.2 ml/ml), basic fibroblast growth factor (1 ng/ml), and insulin (5 µg/ml), otherwise indicated. Additional human lung fibroblasts, LL29, LL97A, CCD‐13Lu, and CCD‐19Lu, were purchased from American Type Culture Collection (ATCC). LL29 and LL97A cells were isolated from 26‐year‐old female and 48‐year‐old male Caucasian IPF patients, respectively. Human normal lung fibroblasts, CCD‐13Lu and CCD‐19Lu, were isolated from a 71‐year‐old male black patient with carcinoma and a 20‐year‐old Caucasian female, respectively. Human normal lung fibroblasts, HLF153 and HLF154, and IPF fibroblasts, IPF12 and IPF14, were kindly provided by Dr. Craig Henke (University of Minnesota) (Bodempudi et al., 2014). Human lung fibroblasts from ATCC and isolated according to the previous report (Bodempudi et al., 2014) were cultured in F12K medium with 10% fetal bovine serum and 1% penicillin–streptomycin. Human embryonic kidney (HEK) epithelial 293T cells (ATCC) were cultured in DMEM with 10% fetal bovine serum and 1% penicillin–streptomycin.

### 2.2 TGFβ and hypoxia treatment

Cells were seeded at a density of 35,000 cells in 35 mm × 10 mm cell culture dishes. Following day, cells were exposed to normoxia (21% O_2_) and hypoxia (1% O_2_) with or without TGFβ1 (5 ng/ml, R & D Systems, Cat. No: 240‐B‐010) for 6 days as previously described (Senavirathna et al., 2018).

### 2.3 RNA isolation and quantitative real‐time PCR

Total RNAs were extracted with TRI Reagent (Molecular Research Institute) according to the manufacturer's instructions. After DNase (Thermofisher Scientific) digestion, cDNAs were synthesized. Then, diluted cDNAs (1:100) were used for real‐time PCR with SYBR green (Eurogentec). Relative gene expression was calculated using 2^−ΔCt^ = 2^−( target Ct‐reference Ct)^. β‐actin was used as the reference gene.

Forward and reverse primers are listed in Table S1. These primers were designed using Prime 3 software (https://www.prime3software.com) and synthesized by Sigma Aldrich. If an lncRNA has more than one transcript, the transcript having the highest lung expression according to the NONCODE (NONCODE2016 version) online database (http://www.noncode.org/) was selected for primer design. Thus, MIR100HG:29 (NONHSAT024781), VCAN‐AS1:6 (NONHSAT102521), TBX2‐AS1:1 (NONHSAT055126) and MRGPRF‐AS1:2 (NONHSAT022561) transcripts were used for primer design. The primers for MIR100HG detect variants 17, 18, 25 and 29. The primers for VCAN‐AS1 detect variant 6. The primers for TBX2‐AS1 detect variant 1. The primers for MRGPRF‐AS1 detect variant 2. For FENDRR, variant 2 was used for primer designing. The primers for FENDRR detect variants 2 and 3.

### 2.4 RNA sequencing analysis

Next‐generation RNA sequencing was performed in three biological replicates on the samples of HPFs treated for 6 days with hypoxia (1% O_2_) and/or TGFβ1 (5 ng/ml) as previously described (More et al., 2019). The RNA sequencing datasets were submitted to GEO (access number: http://www.ncbi.nlm.nih.gov/geo/query/acc.cgi?acc=GSE139963). Briefly, the quality of RNA samples was determined using the Agilent 2100 bioanalyzer. All samples were subjected to polyA enrichment, selection of polyA RNAs using oligo‐dT beads, fragmentation, cDNA synthesis, adaptor ligation, and sequencing using Illumina NextSeq 500 system. Sequencing was completed with one run. Bcl files were converted to FastaQ data after the run. Up to 20 million paired‐end reads for all samples were generated. RNA reads were mapped to genome (GRCh37/hg19) by TopHat2 alignment tool. Differentially expressed mRNAs and lncRNAs were identified using Cuffdiff analysis. mRNAs and lncRNAs having a fold change of ≥2 and a false discovery rate (FDR) value of <0.05 were considered as de‐regulated mRNA and lncRNAs. Gene ontology (GO), disease ontology (DO), and Kyto encyclopedia of genes and genomes (KEGG) pathway analyses were performed by STRING (http://string-db.org/).

### 2.5 Western blot

Whole cell lysates were extracted using a 1X SDS sample buffer containing 0.06 M Tris (pH 6.8), 2.1% (w/v) SDS, 5% (v/v) glycerol, and 1% (v/v) 2‐mercapto‐ethanol. Protein concentration was determined using a D_C_ protein assay kit (Bio‐Rad, Hercules, CA). Proteins were separated on 10% SDS‐PAGE gels. In all, 15 µg and 30 µg of proteins were loaded to detect pSmad2 and pSmad3, and Smad2/3, respectively. Sixty micrograms of proteins were separated on 8% SDS‐PAGE gels for detecting HIF‐1α and 2α proteins. After proteins were transferred to the nitrocellulose membranes, the membranes were blocked with 5% non‐fat milk in Tris‐Buffered Saline with Tween 20 (TBST) buffer and then incubated with primary antibodies at 4°C overnight on a shaker. The following primary antibodies were used: monoclonal mouse anti‐αSMA (1:10,000 dilution, Sigma‐Aldrich Cat. No: SAB1403519), monoclonal mouse anti‐HIF‐1α (1:300 dilution, BD biosciences, Cat. No: 610958), polyclonal anti‐human HIF‐2α (1:500 dilution, Novus biologicals, Cat, No: 10‐122), monoclonal rabbit anti‐pSmad2 (1:500 dilution, Cell signaling, Cat. No: 3108), monoclonal rabbit anti‐pSmad3 (1:500 dilution, Cell Signaling, Cat. No: 9520), monoclonal rabbit anti‐Smad2/3 (1:500 dilution, Cell signaling, Cat. No:8685), and monoclonal mouse anti‐β‐actin (1:3000 dilution, ThermoFisher Scientific, Cat. No: MA5‐15739). The membranes were incubated with horseradish peroxidase‐conjugated goat anti‐rabbit or anti‐mouse secondary antibodies (1:2000–1:3000 dilutions, Jackson Immunoresearch) at room temperature for 1 hr. Then blots were developed using the super signal chemiluminescent substrate (ThermoFisher Scientific) and imaged with an Amersham Imager 600 (GE Healthcare).

### 2.6 lncRNA quantification in cytoplasmic and nuclear fractions

Cytoplasmic and nuclear fractions were separated and isolated using a cytoplasmic and nuclear RNA purification kit (NORGEN Biotek corp.). cDNA was synthesized using random primers from the RNAs isolated from each fraction and diluted 1:20–1:25. Real‐time PCR was performed to detect the expression of lncRNAs. Actin or GAPDH and RNU2 (U2snRNA) were used as positive controls for cytosolic and nuclear RNA, respectively. RNA expression in nucleus and cytosol fractions was calculated using 2^−ct^ and expressed as a percentage value of total (nucleus + cytosol) RNAs.

### 2.7 shRNA lentivirus construction

Human HIF‐1α, HIF‐2α, Smad2, and Smad3 shRNA vectors and lentiviruses were constructed as previously described (Senavirathna et al., 2018). The shRNA sequences for HIF‐1α and HIF‐2α are given in our previous publication (Senavirathna et al., 2018). The shRNA sequences for Smad2 and Smad3 are as follows: Smad2 (NM_001003652.3), GCCTGATCTTCACAGTCATCA (position in coding DNA sequence [CDS], 802–822); and Smad3 (NM_001145102.1), GCAACCTGAAGATCTTCAACA (position in CDS, 1076‐1096).

### 2.8 Gene silencing

HPF cells were seeded at a density of 35,000 cells/well in six‐well plates and then infected with a lentivirus containing a shRNA (Senavirathna et al., 2018) at a multiplicity of infection (MOI) of 100 for 24 hr. Following day, medium was replaced with fresh growth medium and cells were incubated for 6 days at normoxia or hypoxia (1% O_2_) with or without TGFβ1 (5 ng/ml) treatment.

### 2.9 Statistical analysis

Values were presented as the means ± SE. Statistical analysis was performed using GraphPad Prism 7. One‐way or two‐way ANOVA was performed for multiple groups (equal or more than 3), followed by Tukey's multiple comparisons or Fisher's LSD test. The statistical test used in each experiment was given in figure legends. A *p* value <.05 was considered as statistically significant.

## 3 RESULTS

### 3.1 Hypoxia and TGFβ synergistically increase myofibroblast marker expression

**Figure 1:**
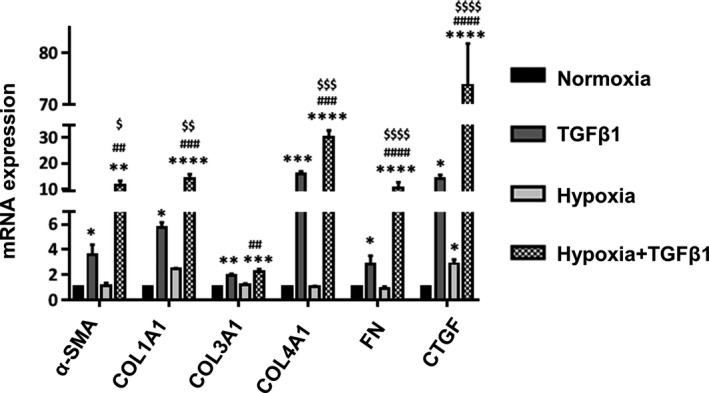
Hypoxia and TGFβ synergistically upregulate myofibroblast marker expression. HPFs were treated with normoxia (21% O_2_), TGFβ1 (5 ng/ml), hypoxia (1% O_2_) or hypoxia (1% O_2_), and TGFβ1 (5 ng/ml) for 6 days. mRNA expression levels of myofibroblast markers were determined by real‐time PCR and normalized to β‐actin. Data were expressed as a fold change to normoxia. Values represent means ± *SE*. *n* = 3 independent experiments. One‐way ANOVA and Tukey's multiple comparison for each gene was performed for statistical analysis. *p < .05, **p < .01, ***p < .001, ****p < .0001 versus normoxia. ^##^ *p* < .01, ^###^ *p* < .001, ^####^ *p* < .0001 versus hypoxia. ^$^ *p* < .05, ^$$^ *p* < .01, ^$$$^ *p* < .001, ^$$$$^ *p* < .0001 versus TGFβ1. αSMA, α‐smooth muscle actin; COL, collagen; CTGF, connective tissue growth factor; FN, fibronectin

To determine the effects of hypoxia and TGFβ on myofibroblast marker expression, HPF cells were exposed to normoxia (21% O_2_), normoxia and TGFβ1, hypoxia (1% O_2_), or hypoxia and TGFβ1 for 6 days. The oxygen concentration in the normal lung tissue is estimated to be 14% and the oxygen level in IPF lung tissue is unknown. However, oxygen levels can reach 0.1% in the severely hypoxic tissue (Bodempudi et al., 2014). The expression of myofibroblast markers including α‐SMA, collagen 1A1, collagen 3A1, collagen 4A1, fibronectin, and CTGF was determined using real‐time PCR. TGFβ1 significantly upregulated the mRNA expression of all the myofibroblast markers in HPFs under the normoxic condition (Figure 1). Hypoxia only significantly increased the mRNA level of CTGF. The combination of hypoxia and TGFβ treatment further upregulated mRNA expression of all the myofibroblast markers except collagen 3A1.

### 3.2 mRNA transcriptome analysis of hypoxia and TGFβ1‐treated human lung fibroblasts

**Figure 2:**
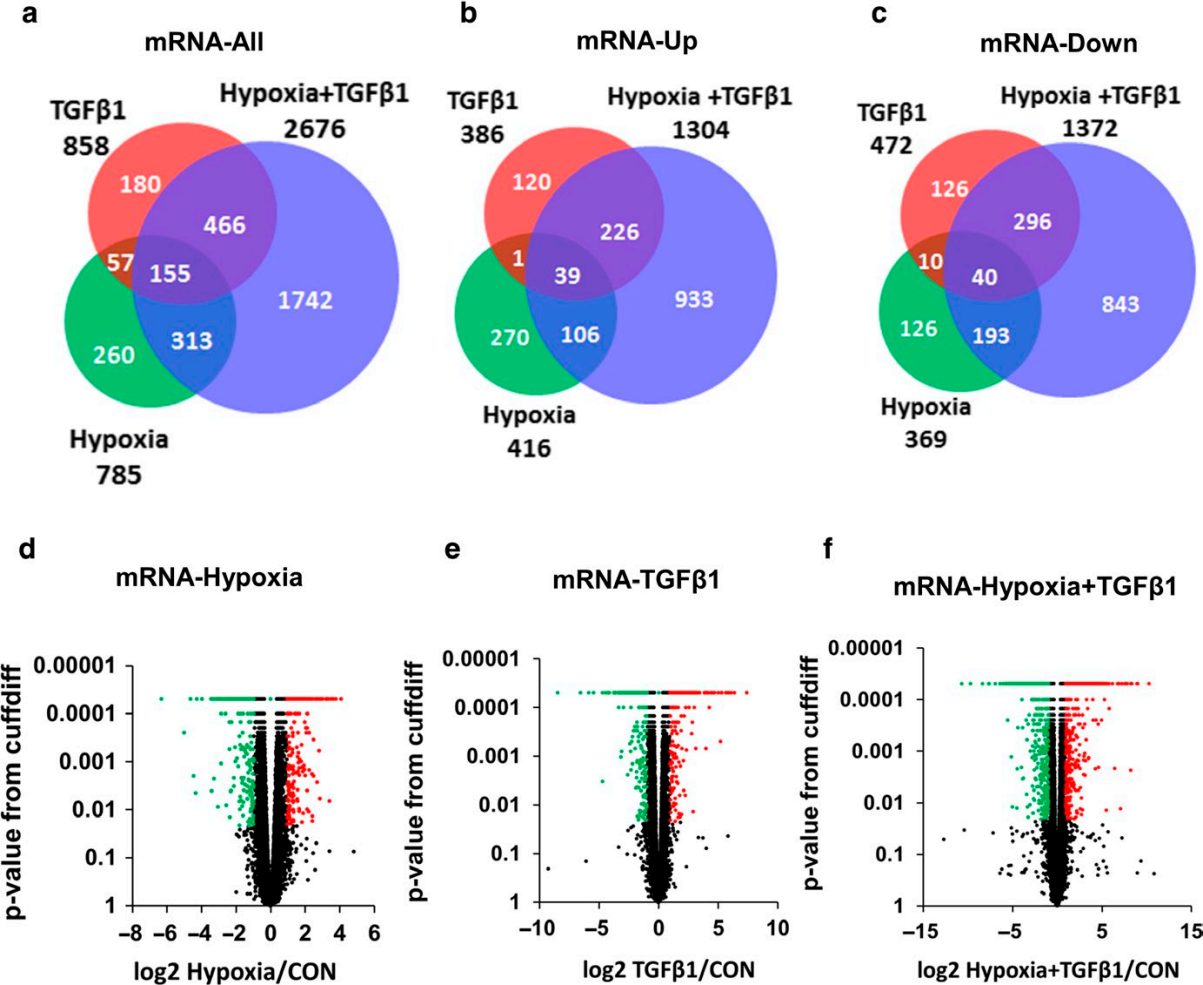
Hypoxia and TGFβ de‐regulate more mRNAs than hypoxia or TGFβ alone in HPF cells. (a–c) Venn diagrams showing the number of significantly de‐regulated mRNAs in HPFs treated with hypoxia, TGFβ1 and hypoxia + TGFβ1 compared to normoxia. (a) All de‐regulated mRNAs. (b) Upregulated mRNAs. (c) Downregulated mRNAs. (d–f) Volcano plots showing the distribution of de‐regulated mRNAs of HPF treated with hypoxia (d), TGFβ1 (e), and hypoxia + TGFβ1 (f). Red and green dots indicate significantly upregulated and downregulated transcripts, respectively (FDR <0.05 and fold change ≥2). Black dots indicate unchanged (fold change below 2) and non‐significant transcripts (FDR ≥0.05). Con: control

To identify mRNAs that were regulated by hypoxia and TGFβ1, we performed RNA sequencing analysis on HPFs treated with hypoxia and/or TGFβ1 for 6 days with three biological replicates. A total of 3,173 mRNAs were de‐regulated by TGFβ1, hypoxia, and hypoxia + TGFβ1 treatments. The numbers of de‐regulated RNAs with a different fold change are listed in the Table S2. The total cumulative number of genes are more than 3,173 in the table because some of the genes are altered by more than one treatment conditions. Of them, 785 (416‐up and 369‐down) mRNAs, 858 (386‐up and 472‐down) mRNAs, and 2,676 (1,304‐up and 1,372‐down) mRNAs were de‐regulated significantly by hypoxia, TGFβ1, and hypoxia + TGFβ1 treatments, respectively, with a fold change of equal or above 2 (Figure 2a–c). Uniquely, there were 260, 180, and 1,742 genes that were de‐regulated only by hypoxia, TGFβ1, and hypoxia + TGFβ1 treatments. In other words, the expression of these genes changed by one treatment was not affected by other two treatments (Figure 2a). It was noted that the number of altered genes in the hypoxia + TGFβ1 group is 6–10 times more than that in hypoxia or TGFβ1 alone group. De‐regulated mRNAs in each treatment group were also represented in volcano plots (Figure 2d–f). Red and green dots indicate upregulated and downregulated mRNAs and black dots represents no changes based on FDR <0.05 and fold change ≥2.

The trend for myofibroblast marker expression including α‐SMA, COL1A1, COL3A1, FN, and CTGF from RNA‐seq data was consistent with the real‐time PCR data (Table S3 and Figure S1), indicating the reliability of RNA‐seq data.

### 3.3 GO analysis shows diverse cellular functions associated with de‐regulated mRNA by hypoxia and TGFβ

GO analysis was performed to associate biological processes, cellular components, and molecular functions with upregulated (Figure S1) and downregulated (Figure S2) mRNAs caused by hypoxia and TGFβ1. Biological processes associated with upregulated mRNAs include cell differentiation, proliferation, signal transduction, and extracellular matrix. Cellular components associated with the upregulated mRNAs are extracellular matrix. Molecular functions of the upregulated mRNAs are mainly involved in protein, iron and kinase binding, indicating the involvement of cellular signaling.

Biological processes of downregulated mRNAs caused by hypoxia + TGFβ1 treatment are cell responses to stimuli, cell communication, regulation of transport, and regulation of cell proliferation. Cellular components associated with the downregulated mRNAs are extracellular region and matrix, extracellular space, and cell projection. Downregulated mRNAs have molecular functions such as protein binding, receptor binding, catalytic activity, co‐factor binding, and transcription factor activity.

**Figure 3:**
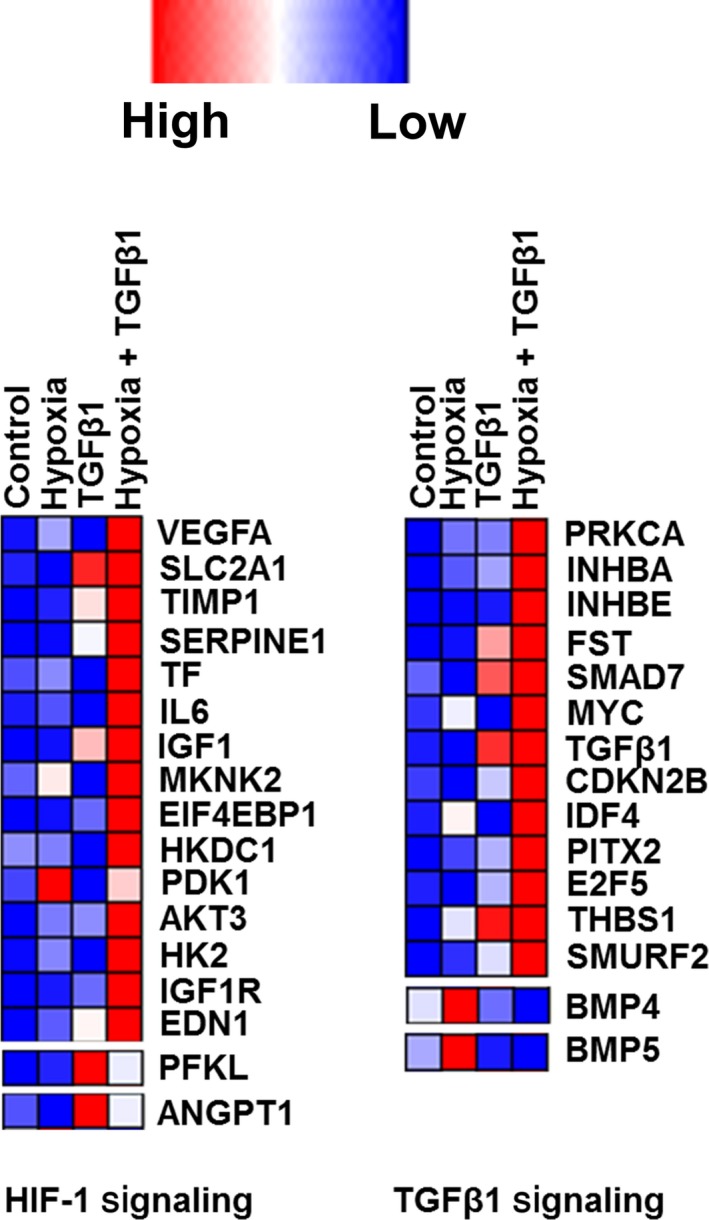
Heat map showing the genes involved in HIF signaling and TGFβ signaling. The color codes from blue to red represent their expression levels from low to high

Using KEGG pathway analysis, we further analyzed signaling pathways regulated by hypoxia, TGFβ1, or their combination (Figure S3). The TGFβ1‐upregulated mRNAs were involved in HIF signaling pathway (*p* value .00281) and the hypoxia‐upregulated mRNAs were involved in TGFβ signaling pathway (*p* value .00392). Upregulated mRNAs by the combinative treatment of hypoxia + TGFβ1 were involved both in HIF signaling (*p* value .0005) and TGFβ signaling (*p* value .00236). These results indicate a cross‐talk between TGFβ and HIF signaling. These genes involved in HIF signaling and TGFβ signaling are represented in a heat map (Figure 3). Hypoxia and TGFβ1 combination treatment upregulated the HIF and TGFβ signaling molecules greater than these treatments alone.

The functions of the genes involved in HIF signaling that are upregulated by TGFβ1 and TGFβ1 + hypoxia are documented in Tables S4 and S5. Most of the TGFβ1‐upregulated genes in HIF signaling are involved in vascular development, angiogenesis, glycolysis, and glucose transport. The hypoxia + TGFβ1‐upregulated genes in HIF signaling have diverse functions ranging from vascular development, glucose transport, and insulin regulation to kinase‐associated phosphorylation.

The functions of the genes involved in TGFβ signaling that were upregulated by hypoxia and hypoxia + TGFβ1 are listed in Tables S6 and S7. Genes involved in TGFβ signaling that were upregulated by hypoxia encode proteins in TGFβ superfamily and adhesive glycoproteins. Genes involved in TGFβ signaling that are upregulated by hypoxia + TGFβ1 encode member proteins in TGFβ superfamily, regulate TGFβ signaling, inhibit cell cycle, or encode transcriptional factors and transcription activators.

### 3.4 Cross‐talk between HIF and TGFβ signaling in human pulmonary fibroblasts

**Figure 4:**
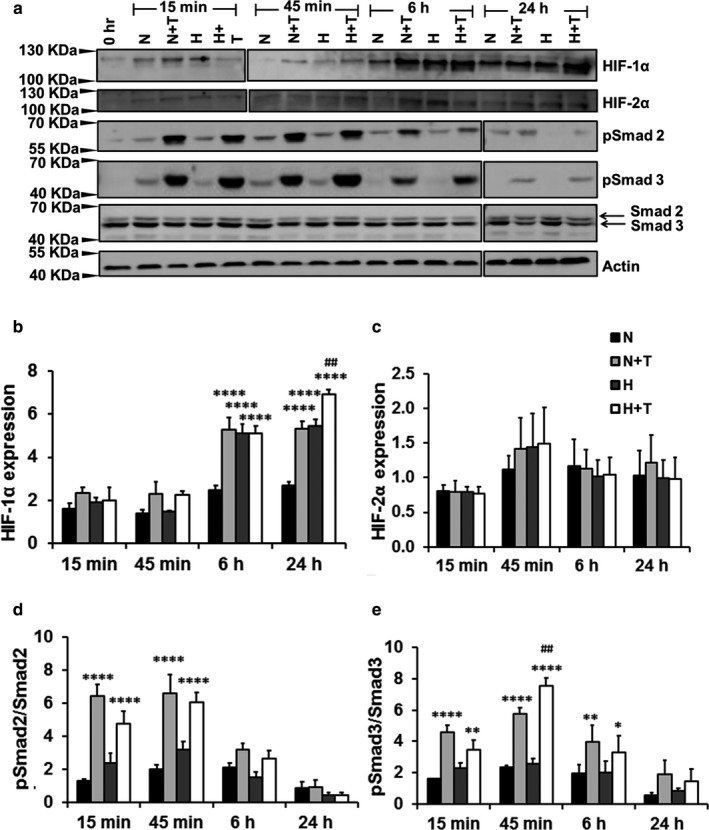
TGFβ1 induces the HIF‐1α expression. Western blots were performed in whole cell lysates of HPFs exposed to normoxia/hypoxia for 3 days, followed by treatment with TGFβ1 and/or hypoxia for 15 min to 24 hr. (a) Western blots. Due to a limited number of wells in SDS gels, two blots, as indicated by two separate borders, for each protein were generated by running two gels at the same time with the same conditions for transfer, antibody incubation and exposure. (b–e) Quantitation of protein expression. Quantification was performed using Image J software. The protein levels of HIF‐α (b) and HIF‐2α (c) were first normalized to β‐actin and then expressed as a ratio to 0 hr. pSmad2 (d) and pSamd3 (e) levels were represented as a ratio of pSmad2 or 3 to total Smad2 or 3. Values represent means ± SE. *n* = 3 independent experiments. Two‐way ANOVA and Fisher's LSD test was performed for statistical analysis. *p < .05, **p < .01, ****p < .0001 versus normoxia. ^##^ *p* < .001 versus normoxia + TGFβ1. N: Normoxia (21% O_2_), T: TGFβ1 (5 ng/ml), H: Hypoxia (1% O_2_), H + T: hypoxia (1% O_2_) + TGFβ1 (5 ng/ml)

To confirm the cross‐talk between HIF and TGFβ signaling, we examined the effects of hypoxia and TGFβ1 on HIF‐1α and HIF‐2α protein expression and phosphorylated Smad2 and 3. HPFs were exposed to hypoxia or normoxia for 3 days and then treated with TGFβ1 and hypoxia for up to 24 hr. HIF‐1α protein expression was markedly upregulated by TGFβ1 at 6 hr and 24 hr under normoxic conditions and further enhanced by a combination of hypoxia and TGFβ1 at 24 hr (Figure 4a,b). However, TGFβ1 had no effects on HIF‐2α protein levels under normoxic or hypoxic conditions. The absence of hypoxia‐induced HIF1α expression in the cells previously exposed to normoxia is likely due to the short exposure of hypoxia (24 hr or less). Phosphorylated Smad 2 and 3 were increased by TGFβ1 starting at 15 min and the signal was faded after 6 hr (Figure 4a,d,e). Hypoxia did not affect phosphorylated Smad 2 or 3 level in the presence or absence of TGFβ1 except that a combination of hypoxia and TGFβ1 increased phosphorylated Smad 3 level at 45 min compared to TGFβ1 alone (Figure 4a,e).

### 3.5 LncRNA transcriptome analysis of hypoxia and TGFβ‐treated human pulmonary fibroblasts

**Figure 5:**
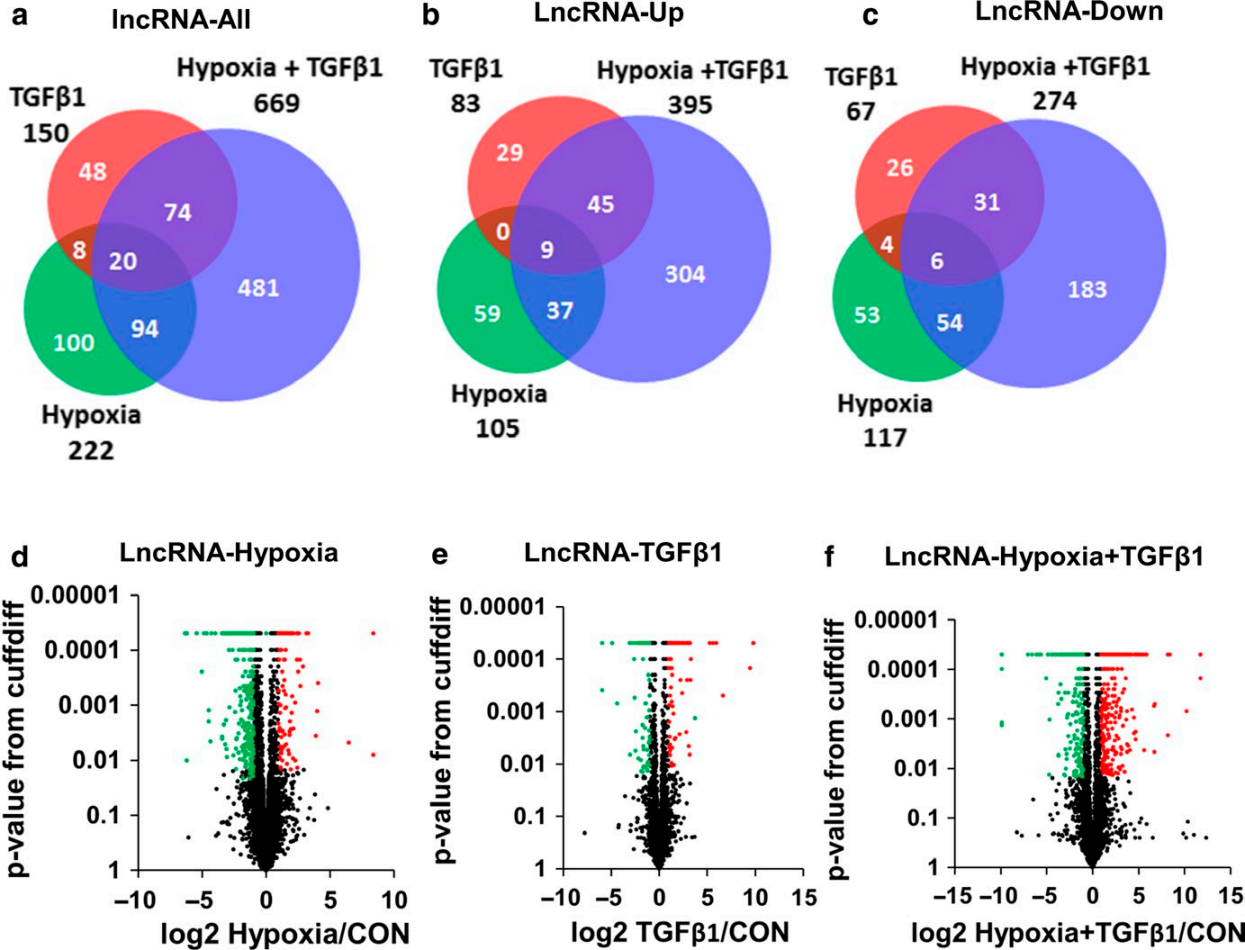
LncRNAs are de‐regulated in HPF cells treated with hypoxia and TGFβ1. (a–c) Venn diagram showing the number of significantly de‐regulated lncRNAs with a fold change of ≥2 of HPFs treated with hypoxia (1% O_2_), TGFβ1 and hypoxia + TGFβ1 compared to normoxia control. (a) De‐regulated all lncRNAs (both upregulated and downregulated). (b) Upregulated lncRNAs. (c) Downregulated lncRNAs. (d–f) Volcano plots showing the distribution of de‐regulated lncRNAs of HPFs treated with hypoxia (d), TGFβ1 (e), and hypoxia + TGFβ1 (f) compared to the control. Red and green dots indicate significantly upregulated and downregulated transcripts, respectively (FDR <0.05 and fold change ≥2). Black dots indicate unchanged (fold change below 2) and non‐significant transcripts (FDR ≥0.05). CON, control

We further analyzed RNA sequencing data of HPFs treated with hypoxia (1% O_2_) and/or TGFβ1 for 6 days as these used for mRNA transcriptome analysis to identify hypoxia/TGFβ1‐regulated lncRNAs. A total of 825 lncRNAs were significantly de‐regulated with a fold change of ≥2 by TGFβ1, hypoxia, and/or hypoxia + TGFβ1 (Figure 5a). Among them, the de‐regulated lncRNAs were 222 (105‐up and 117‐down) in the hypoxia group, 150 (83‐up and 67‐down) in the TGFβ1 group, and 669 (395‐up and 274‐down) in the hypoxia + TGFβ1 group (Figure 5b,c). The expression of 100, 48, and 481 lncRNAs was altered by only one treatment condition, hypoxia, TGFβ1, and hypoxia + TGFβ1, respectively. Hypoxia + TGFβ1 treatment shows a larger number of upregulated and downregulated lncRNAs compared to each treatment alone, which is consistent with the de‐regulated mRNAs. The numbers of lncRNAs with a different fold change are listed in Table S8. De‐regulated lncRNAs in each treatment group are also represented in volcano plots (Figure 5d–f). The red and green dots indicate significantly upregulated and downregulated lncRNAs (FDR <0.05 and fold change ≥2) and the black dots represent the lncRNAs having a fold change <2 and FDR ≥0.05.

### 3.6 GO analysis shows diverse cellular functions associated with the neighboring genes of de‐regulated lncRNAs

As the functions of most lncRNAs are unknown and lncRNAs are predicted to regulate their neighboring genes (Vance & Ponting, 2014), we selected neighboring genes within 1,000 kb distance from the de‐regulated lncRNAs and performed GO analysis. Biological processes associated with neighboring genes of upregulated lncRNAs by TGFβ1 and hypoxia are extracellular matrix organization, regulation of protein kinase activity, and protein phosphorylation (Figure S4a). Cellular components of neighboring genes of upregulated lncRNAs are collagen and extracellular matrix (Figure S4b). Molecular functions of neighboring genes of upregulated lncRNAs are involved in fibroblast growth factor, collagen, and fibronectin binding (Figure S4c). Biological processes associated with neighboring genes of downregulated lncRNAs by TGFβ1 and hypoxia were mainly associated with immunity including innate and adaptive immune responses (Figure S5a). Cellular components of neighboring genes of downregulated lncRNAs were mostly involved in intracellular organelles such as phagocytic vesicle and lysosomal membrane, endosome, and nucleus (Figure S5b). Molecular functions of downregulated lncRNAs are protease and DNA binding (Figure S5c).

Disease ontology (DO) analysis showed that TGFβ1 and hypoxia‐de‐regulated lncRNAs were associated with many diseases including IPF and pneumonia. Among them, IPF ranked on the top with a *p* value of .0001 (Figure S6).

### 3.7 Selection and validation of the identified lncRNAs

We selected five upregulated and five downregulated lncRNAs in the hypoxia + TGFβ1 group for further studies using the following selection criteria: (a) a fold change of >5. In all, 127 upregulated and 77 downregulated lncRNAs met this criterion. (b) The basal expression level of fragments per kilobase of exon per million fragments mapped (FPKM) value of above 10 for the upregulated lncRNAs and above 50 for the downregulated lncRNAs. In all, 36 upregulated and 34 downregulated lncRNAs met this criterion. (c) The lncRNAs expression levels in the lungs obtained from NONCODE (NONCODE2016 version) online database (http://www.noncode.org/). lncRNAs having NONCODE expression above 1 were selected and 14 and 15 lncRNAs in upregulated and downregulated groups met this criterion. (d) Finally, five upregulated and five downregulated lncRNAs having the highest basal expression level (FPKM) were selected for validation. The number of transcripts, size, locus, FPKM expression, fold change with hypoxia and TGFβ1 treatment and NONCODE database lung expression of those selected lncRNAs were listed in Table S9.

**Figure 6:**
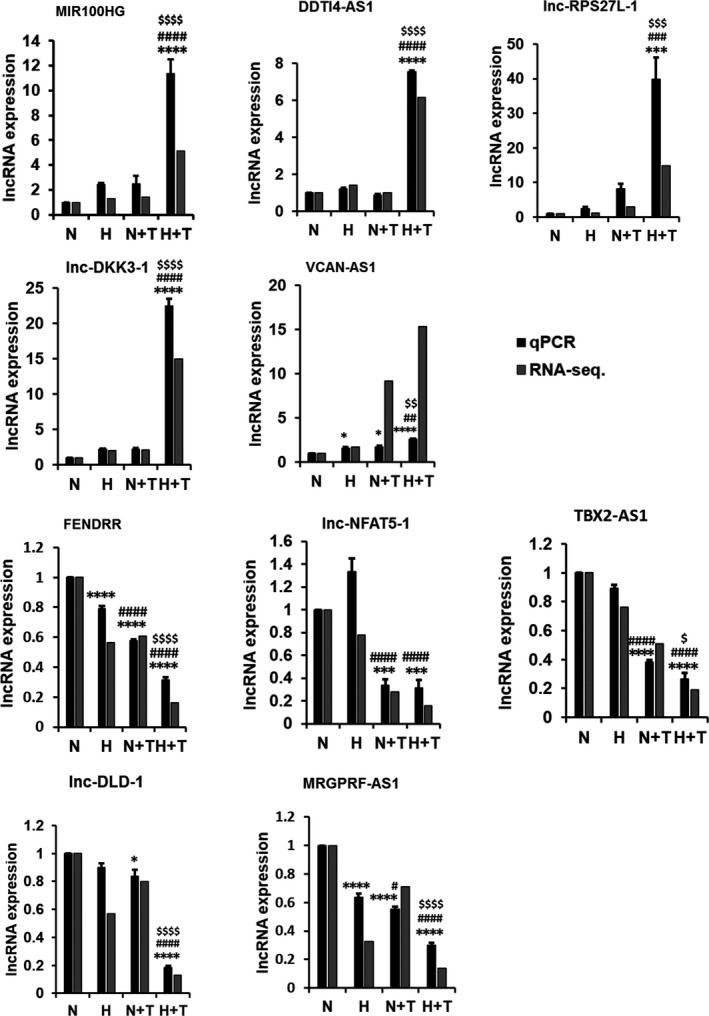
Real‐time PCR validation of RNA sequencing data of upregulated and downregulated lncRNAs in hypoxia and/or TGFβ1 treatment. HPF cells were treated with hypoxia (1% O_2_) and/or TGFβ1 (5 ng/ml) for 6 days. Real‐time PCR was conducted to detect lncRNA expression. Data were normalized to β‐actin and then normalized to 21% O_2_ normoxia control. RNA‐seq data were represented as fold change compared to normoxia group. Values represent means ± *SE*. *n* = 3 independent experiments. One‐way ANOVA and Tukey's multiple comparison was performed for statistical analysis. *p < .05, ***p < .001, ****p < .0001 versus normoxia.^ #^ *p* < .05, ^##^ *p* < .01, ^###^ *p* < .001, ^####^ *p* < .0001 versus hypoxia, ^$^ *p* < .05, ^$$^ *p* < .01, ^$$$^ *p* < .00, ^$$$$^ *p* < .0001 versus TGFβ1. (N: Normoxia, T: TGFβ, H: Hypoxia, H + T: Hypoxia + TGFβ1)

The selected lncRNAs were validated by real‐time PCR (Figure 6). The trend in changes from RNA sequencing and real‐time PCR data were similar except VCAN‐AS1 although there were some variations in fold changes (e.g., MIR100HG and lnc‐RPS27L‐1).

### 3.8 Subcellular localization of lncRNAs

**Figure 7:**
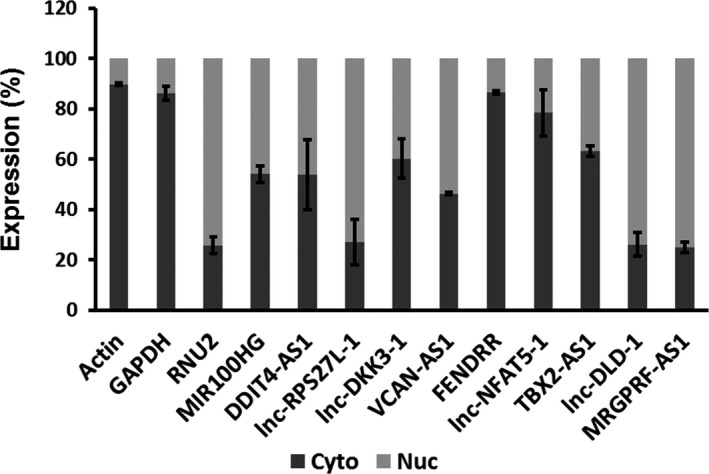
Cytosolic and nuclear levels of selected lncRNAs. Nuclear and cytosolic fractions were isolated from HPFs and real‐time PCR was performed to detect the lncRNA expression. Data were represented as a percentage of the total expression. Values represent means ± *SE*. *n* = 3 independent experiments

We determined the subcellular localization of the validated lncRNAs by extracting RNAs from cytoplasm and nuclear fractions of HPFs and performing real‐time PCR analysis. Actin/GAPDH and U2 were used as positive controls for cytoplasm and nucleus, respectively. FENDRR, and lnc‐NFAT5‐1 were enriched in cytosol and lnc‐RPS27L‐1, lnc‐DLD‐1, and MRGPRF‐AS1 were enriched in nucleus (Figure 7). MIR100HG, DDIT4‐AS1, lnc‐DKK3‐1, VCAN‐AS1, and TBX2‐AS1 were distributed in both cytoplasm and nucleus.

### 3.9 FENDRR is downregulated by hypoxia and TGFβ1

**Figure 8:**
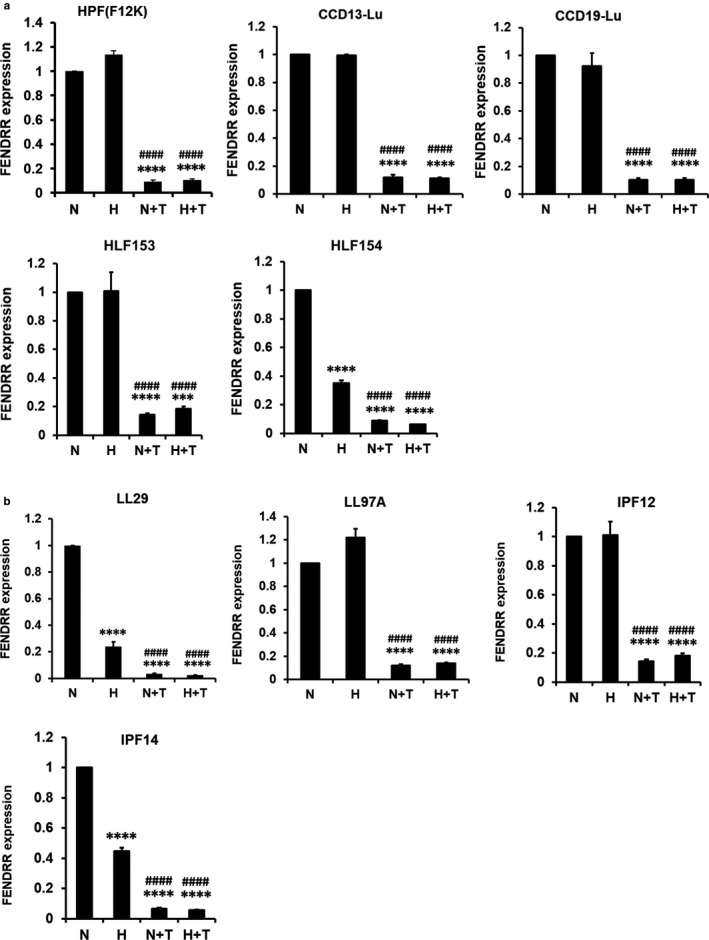
Effects of hypoxia and TGFβ1 on FENDRR expression in different human pulmonary fibroblasts. Pulmonary fibroblasts isolated from the lungs of normal subjects (a) and IPF patients (b) were cultured F12K medium containing 10% FBS and were treated with normoxia (21% O_2_), hypoxia (1% O_2_), normoxia + TGFβ1 (5 ng/ml), and hypoxia (1% O_2_) + TGFβ1 (5 ng/ml) for 6 days. FENDRR expression was determined by real‐time PCR and normalized to β‐actin. Data were expressed as a ratio to normoxia. Values represent means ± *SE*. *n* = 3 independent experiments. One‐way ANOVA and Tukey's multiple comparison were performed for statistical analysis. ****p < .0001 versus normoxia. ^####^ *p* < .0001 versus hypoxia. N: normoxia, H: hypoxia, N + T: normoxia + TGFβ1, H + T: hypoxia + TGFβ1

FENDRR is chosen for further studies due to its importance in development (Grote & Herrmann, 2013; Grote et al., 2013). We examined the effects of culture media and multiple fibroblast lines on hypoxia and TGFβ1‐mediated regulation of FENDRR expression. We cultured HPFs in fibroblast medium (PromoCell) with the supplements in the all of the experiments performed so far. To determine the culture medium effects, we cultured HPFs in F12K medium plus 10% FBS, which are used for most of lung fibroblast culture. TGFβ1 and/or hypoxia downregulated FENDRR expression in the F12K medium more than the cells grown in PromoCell commercial medium (Figure 8).

The downregulation of FENDRR by TGFβ1 was consistently observed in other four normal human fibroblast lines (CCD13‐Lu, CCD19‐Lu, HLF153, and HLF154) and four IPF lung fibroblast lines (LL29, LL97A, IPF12, and IPF14), all cultured in F12K medium. However, hypoxia did not cause further reduction of FENDRR expression, probably because TGFβ1 already reduces the FENDRR expression to the lowest level. The effects of hypoxia on FENDRR expression in human lung fibroblasts cultured on F12K medium were variable and hypoxia only reduced the FENDRR expression in three of nine fibroblast lines (HLF154, LL29, and IPF 14).

### 3.10 Silencing of HIF‐1α and Smad3 rescues the downregulation of FENDRR by hypoxia and TGFβ1

**Figure 9:**
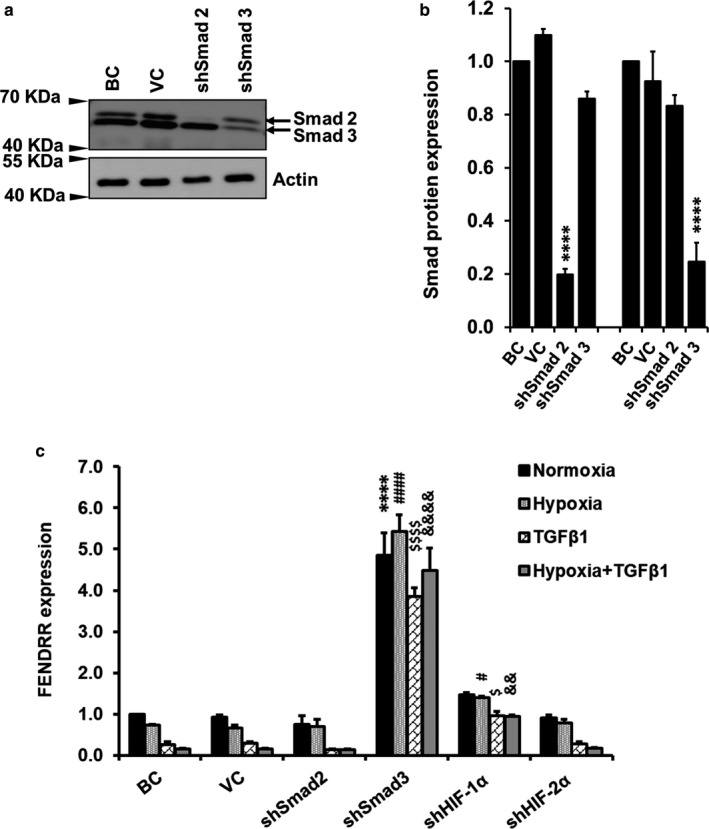
HIF‐1α and Smad3 silencing rescues the hypoxia and TGFβ1‐mediated FENDRR downregulation. HPFs were infected with shRNA lentiviral constructs and treated with TGFβ1 and hypoxia for 6 days. (a) Western blot showing Smad2 and Smad3 silencing. (b) Quantification of protein expression as performed with image J software. Data were normalized to β‐actin and then normalized to blank control (BC). Values represents means ± *SE*. *n* = 3 independent experiments. Two‐way ANOVA and Tukey's test was performed for statistical analysis. ****p < .0001 versus VC. (c) FENDRR expression was determined by real‐time PCR and normalized to β‐actin. Data were expressed as a fold change to blank control (BC) at normoxia. Values represent means ± SE. *n* = 3 independent experiments. Two‐way ANOVA and Fisher's LSD test were performed for statistical analysis. ****p < .0001 versus VC‐Normoxia. ^#^ *p* < .05, ^####^ *p* < .0001 versus VC‐Hypoxia. ^$^ *p* < .05 ^$$$$^ *p* < .0001 versus VC‐TGFβ1. ^&&^ *p* < .01, ^&&&&^ *p* < .0001 versus VC‐Hypoxia + TGFβ1. VC, vector control

To determine which HIF and Smad isoforms may participate in hypoxia and TGFβ1‐mediated downregulation of FENDRR, we silenced HIF‐1α, HIF‐2α, Smad2, or Smad3 using shRNA lentiviral constructs as previously described (Senavirathna et al., 2018). The silencing efficiencies of HIF‐1α and HIF‐2α were given in the previous publication under the same conditions and the same type of HPF cells (Senavirathna et al., 2018). The silencing efficiencies of Smad2 and Smad3 are shown in Figure 9a,b. Silencing of Smad3 and HIF‐1α resulted a higher FENDRR expression than vector control or blank control under all of the treatment conditions (normoxia, TGFβ1, hypoxia, or TGFβ1 + hypoxia) (Figure 9c). However, the observed effect was much greater in Smad3 silencing than HIF‐1α silencing. Silencing of Samd2 or HIF‐2α had no effects on FENDRR expression. These results indicate that Smad3 and HIF‐1α could be involved in the FENDRR downregulation by TGFβ1 and hypoxia.

## 4 DISCUSSION

Fibroblast proliferation and differentiation are the major contributors to excess extracellular matrix production, a prominent feature of IPF. While hypoxia is one of the key factors for rapid fibroblast proliferation, TGFβ signaling promotes the differentiation of fibroblasts to myofibroblasts. In this study, we found that TGFβ1 and hypoxia synergistically increased the mRNA expression of myofibroblast markers in HPFs. RNA‐seq analyses further revealed that the combination of hypoxia and TGFβ1 treatment caused much more changes in mRNA and lncRNA expression in HPFs than hypoxia or TGFβ1 treatment alone.

It is known that TGFβ is a key regulator in fibroblast differentiation in fibrotic disorders in kidney, lung, heart, liver, and other organs (Bondi et al., 2010; Khalil et al., 2017; Pohlers et al., 2009; Thannickal et al., 2003; Webber, Jenkins, Meran, Phillips, & Steadman, 2009; Yi et al., 2014). Upon TGFβ stimulation, fibroblasts differentiate into myofibroblasts, which express myofibroblast markers such as collagen 1, α‐SMA, fibronectin, and connective tissue growth factor (CTGF) (Klingberg, Hinz, & White, 2013). Our current finding that hypoxia increased TGFβ1‐mediated myofibroblast marker expression suggests that hypoxia may enhance TGFβ1‐mediated fibroblast differentiation. This conclusion is supported by several previous studies. It is reported that a 2‐day hypoxia exposure enhances TGFβ‐induced collagen I expression and secretion in human lung fibroblasts (Papakonstantinou, Aletras, Roth, Tamm, & Karakiulakis, 2003). Another study shows that a 2‐day hypoxia exposure increases the expression of α‐SMA in cardiac fibroblasts (Gao, Chu, Hong, Shang, & Xu, 2014). Furthermore, the exposure of pulmonary fibroblasts to hypoxia for 8 days results in an increase in α‐SMA, collagens I and III expression (Robinson, Neary, Levendale, Watson, & Baugh, 2012).

A large number of mRNAs and lncRNAs were de‐regulated by a combination of hypoxia and TGFβ1 treatment compared to hypoxia or TGFβ1 alone, suggesting an interaction between these two signaling pathways. Our KEGG pathway analysis showed that hypoxia upregulated the genes in TGFβ signaling and vice versa. Several previous studies support cross‐talk between hypoxia and TGFβ signaling pathways in lung fibroblasts and other cells. The inhibition of HIF‐1 reduces the TGFβ expression in human fetal lung fibroblasts (Qian et al., 2015). Hypoxia stimulates the TGFβ secretion in lung fibroblasts and endothelial cells (Papakonstantinou et al., 2002; Zhang et al., 2003). These results are consistent with our RNA‐seq data showing that hypoxia increases the expression of several members of the TGFβ family such as TGFβ1, BMP4, and BMP5. Thrombospondin 1 is also upregulated by hypoxia treatment in our RNA‐seq data set and this gene is implicated in the activation of latent TGFβ and promotes tissue fibrosis (Sakai et al., 2003; Sweetwyne & Murphy‐Ullrich, 2012).

Our current studies indicated that TGFβ1 enhanced the hypoxia‐induced HIF‐1 protein expression in human adult lung fibroblasts. It is reported that TGFβ induces the HIF‐1α protein in human embryonic lung fibroblasts and increases hypoxia responsive element–luciferase reporter activity (Abdul‐Hafez et al., 2009). TGFβ also stimulates HIF‐1α accumulation in mouse alveolar macrophages (Ueno et al., 2011).

Our RNA‐seq data showed that TGFβ1 and hypoxia upregulated the genes involved in the HIF signaling, including SLC2A1 (GLUT‐1), SERPINE1 (PAI‐1), phosphofructokinase‐(L), IGF1, EIF4EBP1, endothelin 1, and angiopoietin 1. Some genes such as GLUT‐1, phosphofructokinase‐(L), PAI‐1, and angiopoietin‐1 are reported to be upregulated by hypoxia (Liu, Moller, Flugel, & Kietzmann, 2004; Minchenko, Opentanova, & Caro, 2003; Semenza, 2012; Zhang, Behrooz, & Ismail‐Beigi, 1999). GLUT‐1 and phosphofructokinase‐(L) are involved in glucose transport (Minchenko et al., 2003; Zhang et al., 1999) while PAI‐1 and angiopoietin‐1 are involved in angiogenesis (Liu et al., 2004; Semenza, 2012). GLUT‐1 and phosphofructokinase are known to be induced in fibrotic lungs as metabolic dysregulation is a common feature in IPF (Andrianifahanana et al., 2016; Zank, Bueno, Mora, & Rojas, 2018). TGFβ also induces angiogenesis in IPF which is evident in pulmonary hypertension associated with IPF (Farkas, Gauldie, Voelkel, & Kolb, 2011). IGF‐1 and endothelin‐1 induce HIF‐1 protein expression (Fukuda et al., 2002; Li et al., 2012; Zelzer et al., 1998). IGF‐1 and endothelin‐1 are growth factors which are mainly involved in fibroblast proliferation in IPF (Allen & Spiteri, 2002). EIF4EBP1 is the inhibitory regulator of translation initiation factor EIF4E. Hypoxia induces the expression of EIF4RBP1 to shut‐off cellular protein synthesis (Tinton & Buc‐Calderon, 1999).

In this study, we identified 825 lncRNAs that were de‐regulated by hypoxia and TGFβ1. Based on gene ontology analyses, the neighboring genes of these altered lncRNAs have functions associated with extracellular matrix structure, collagen production, and collagen and fibronectin binding, suggesting that these lncRNAs likely play important roles in the pathogenesis of IPF. There are very limited studies on the roles of lncRNAs in lung fibrosis. We have reported that silencing of lncRNA CD99 molecule pseudogene 1 (CD99P1) inhibits cell proliferation and α‐SMA expression in lung fibroblasts while silencing of lncRNA n341773 reduces collagen expression in these cells (Huang et al., 2015). LncRNAs AJ005396 and S69206 are upregulated in the lungs of a rat model of bleomycin‐induced fibrosis and located in the cytoplasm of interstitial lung cells (Cao et al., 2013). LncRNAs uc.77 and 2700086A05Rik are found to be important regulators in epithelial mesenchymal transition (EMT) in paraquet induced‐lung fibrosis in mice. Overexpression of these lncRNAs in epithelial cells induces the mesenchymal marker expression (Sun et al., 2016).

The subcellular localization of an lncRNA may provide information as regards to how an lncRNA functions in cells. We observed that FENDRR was mainly located in the cytoplasm of normal lung fibroblasts, which is consistent with its cytoplasmic location in MG63 and KH‐OS human osteosarcoma cells (Kun‐Peng & Xiao‐Long, 2017). While we found that MIR100HG was equally distributed in cytoplasm and nucleus in normal lung fibroblasts, MIR100HG is predominantly located in the nucleus of human megakaryotic leukemia cells (CMK) (Emmrich et al., 2014) and osteosarcoma (U2OS) cells (Sun et al., 2018). Thus, an lncRNA may have a different cellular location in different types of cells. Our data show an equal distribution of DDIT4‐AS1, Lnc‐DKK3‐1, VCAN‐AS1 in cytoplasm and nucleus, nuclear localization of lnc‐RPS27L‐1, lnc‐DLD‐1, MRGPRF‐AS1 and cytosol localization of lnc‐NFAT5‐1, TBX2‐AS1 in normal lung fibroblasts. There are no reports of subcellular localization of these lncRNAs. It remains to be determined whether subcellular location of above‐mentioned lncRNAs alter in IPF lungs compared to normal lungs.

Among the downregulated lncRNAs by hypoxia and TGFβ1, FENDRR was most potently and consistently regulated by TGFβ1 in all of the human lung fibroblasts tested. However, the downregulation of FENDRR by hypoxia was only observed in three of nine lung fibroblasts. The regulation of FENDRR expression appears to be dependent on culture medium, likely due to the factors present in the different medium. The mechanisms for the FENDRR regulation by hypoxia and TGFβ1 may involve HIF‐1α and Smad3.

FENDRR is named FOXF1 adjacent non‐coding developmental regulatory RNA because its importance in gene regulatory network in mammalian embryogenesis (Dey, Mueller, & Dutta, 2014). FENDRR is located at chromosome 16 and transcribed in an antisense manner as its neighboring gene, FOXF1 (Herrera et al.,). It causes the methylation of the promoter sites of target genes, and represses their expression via binding polycomb repressive complex 2 (PRC2) and Trithorax group/MLL complex (TRxG/MLL) (Grote & Herrmann, 2013). FENDRR is specifically expressed in nascent lateral phase mesoderm. It is important for heart and body wall development. Loss of fendrr in mice impairs the differentiation of tissue in lateral mesoderm, resulting in defective development of heart and body wall (Grote & Herrmann, 2013; Grote et al., 2013; Sauvageau et al., 2013).

FENDRR is also associated with cancers (Herrera et al.,; Kun‐Peng, Chun‐Lin, & Xiao‐Long, 2017; Xu et al., 2014; Zheng, Krishnan, Zou, & Ongkeko, 2016). The expression of FENDRR is lower in gastric cancer cell lines than that in normal gastric epithelial cells. FENDRR overexpression in gastric cancer cells inhibits cell invasion and migration with a reduced expression of fibronectin 1, metalloproteinase 2 and 9 (Xu et al., 2014). As FENDRR is regulated by hypoxia and TGFβ, two key factors for IPF pathologies, FENDRR likely plays a role in IPF.

In summary, hypoxia and TGFβ treatment synergistically activate myofibroblast markers and de‐regulate a larger number of mRNAs and lncRNAs than hypoxia or TGFβ1 alone. FENDRR is likely regulated via Smad3 and HIF‐1α.
