# Supplementary figures and images for: Hypoxia and transforming growth factor β1 regulation of long non‐coding RNA transcriptomes in human pulmonary fibroblasts
Source: Physiol Rep. 2020 Jan 10;8(1):e14343. doi: 10.14814/phy2.14343 (PMC6954122; doi:10.14814/phy2.14343)

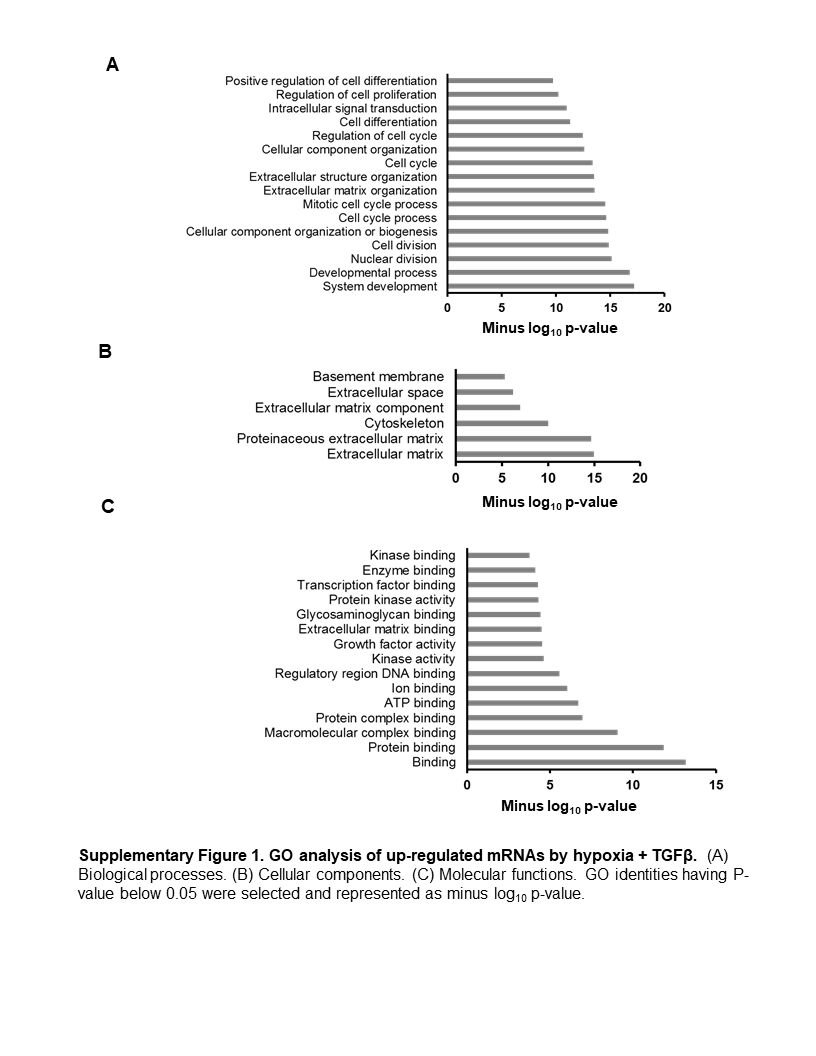

Supplement: Supplementary file 1 [file PHY2-8-e14343-s001.TIF]

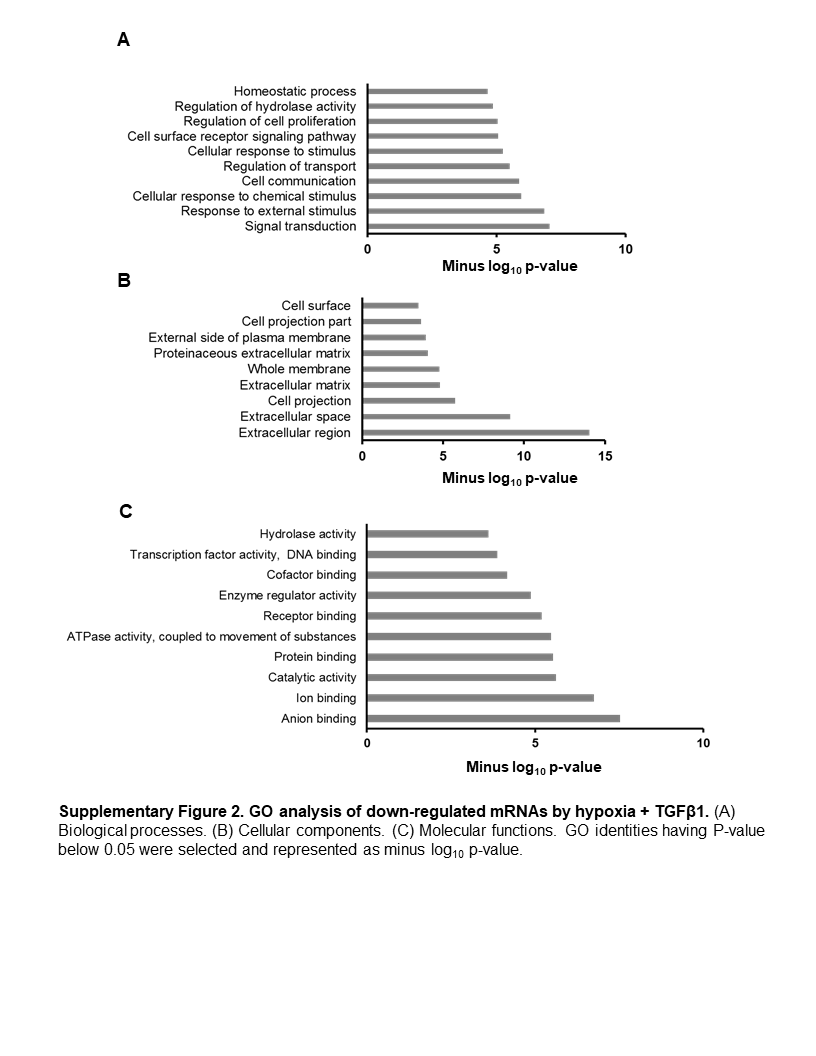

Supplement: Supplementary file 2 [file PHY2-8-e14343-s002.TIF]

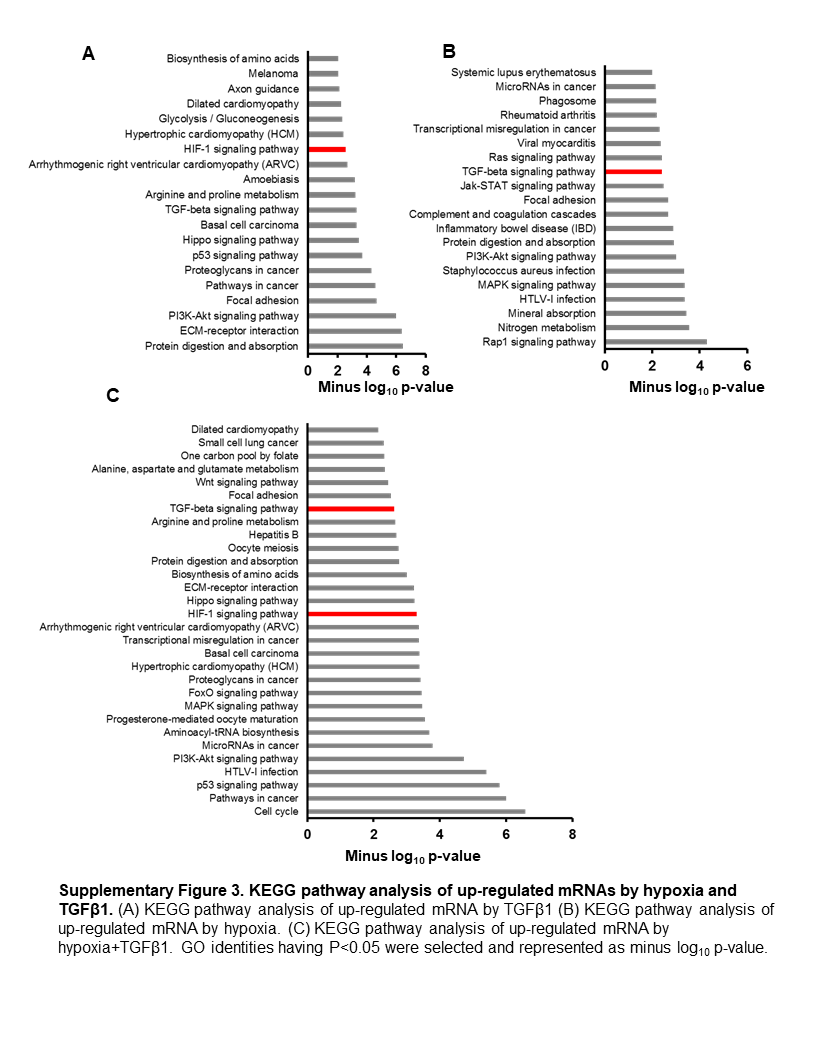

Supplement: Supplementary file 3 [file PHY2-8-e14343-s003.TIF]

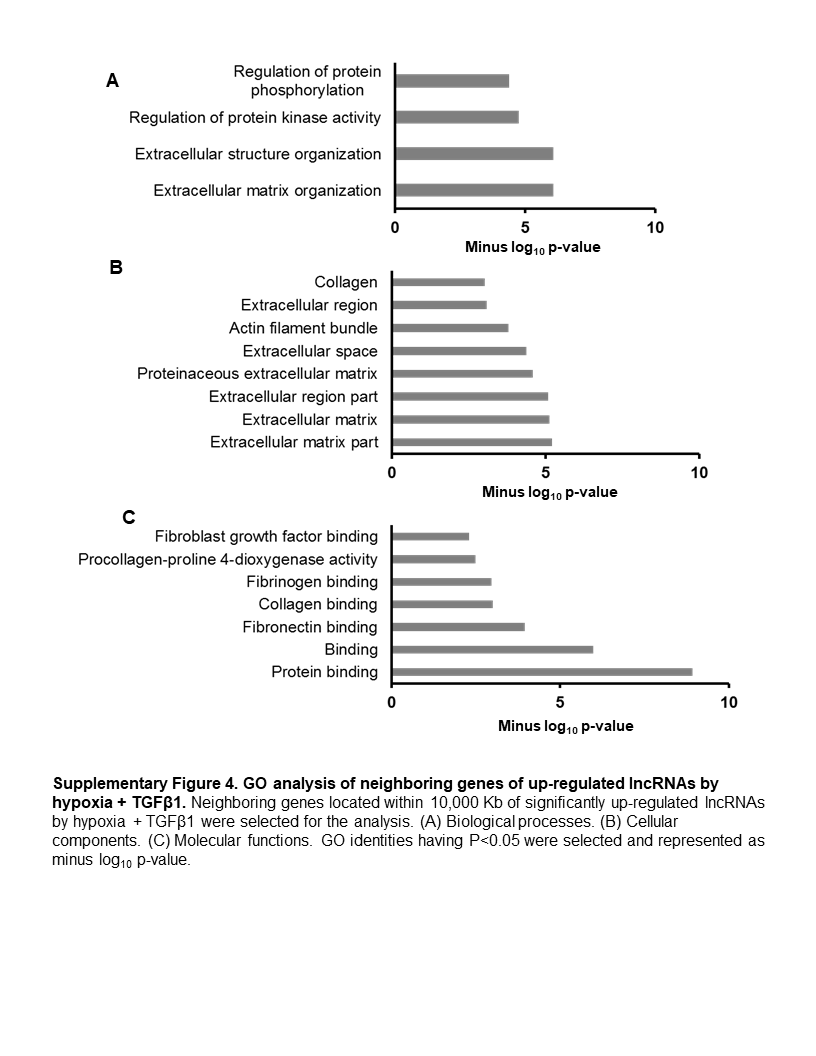

Supplement: Supplementary file 4 [file PHY2-8-e14343-s004.TIF]

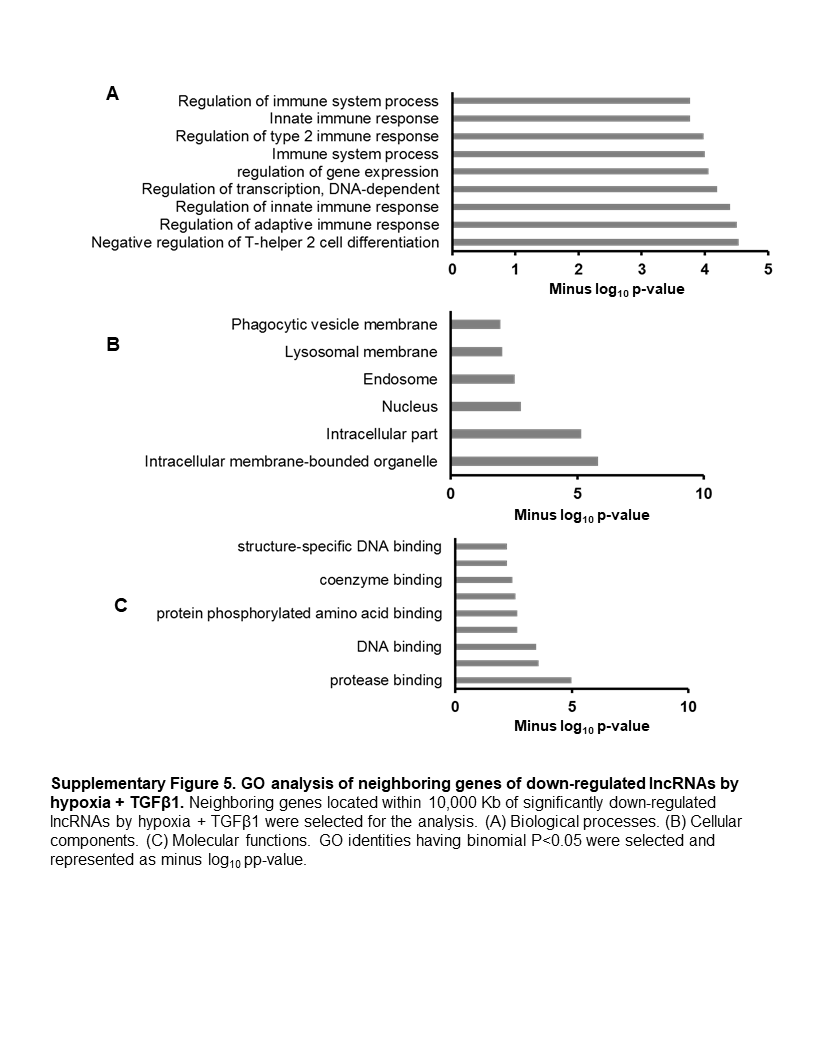

Supplement: Supplementary file 5 [file PHY2-8-e14343-s005.TIF]

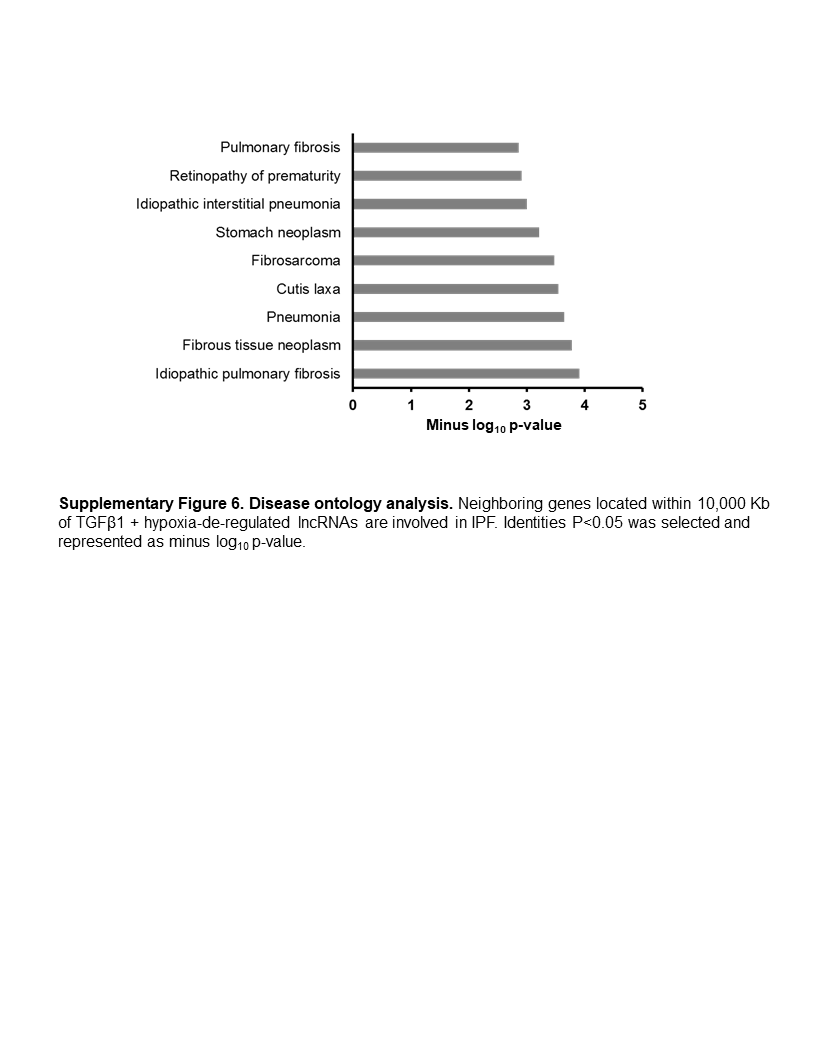

Supplement: Supplementary file 6 [file PHY2-8-e14343-s006.TIF]
