# Supplementary material for: Hypoxia and transforming growth factor β1 regulation of long non‐coding RNA transcriptomes in human pulmonary fibroblasts
Source: Physiol Rep. 2020 Jan 10;8(1):e14343. doi: 10.14814/phy2.14343 (PMC6954122; doi:10.14814/phy2.14343)
Supplement: Supplementary file 7 [file PHY2-8-e14343-s007.docx]

**A**

**KEGG: TGFβ-Up regulated**

**B**

**KEGG: Hypoxia-Up regulated**

**C**

**KEGG: Hypoxia+TGF β-Up regulated**

**A**

| **Supplementary Table 1: Primers used for real-time PCR** | | |  |
| --- | --- | --- | --- |
| α-SMA-FW |  | GAGAAGAGTTACGAGTTGCCTGA | |
| α-SMA-RE |  | TGTTAGCATAGAGGTCCTTCCTG | |
| Collagen 1A1-FW |  | CGAAGACATCCCACCAATCAC | |
| Collagen 1A1-RE |  | CAGATCACGTCATCGCACAAC | |
| Collagen 3A1-FW |  | TGGCTACTTCTCGCTCTGCTT |  |
| Collagen 3A1-RE |  | TTCCAGACATCTCTATCCGCATAG |  |
| Collagen 4A1-FW |  | CTCTGGCTGTGGCAAATGTG |  |
| Collagen 4A1-RE |  | CCTCAGGTCCTTGCATTCCA |  |
| Fibronectin-FW |  | CCTGCATCTGAGTACACCGTATC |  |
| Fibronectin-RE |  | GGTCTCAGTCACCTCGGTGTT |  |
| CTGF-FW |  | CAGCATGGACGTTCGTCTG |  |
| CTGF-RE |  | AACCACGGTTTGGTCCTTGG |  |
| MIR100HG-FW  MIR100HG-RE  DDTI4-AS1-FW  DDTI4-AS1-RE  lnc-RPS27L-1-FW  lnc-RPS27L-1-RE  lnc-DKK3-1-FW  lnc-DKK3-1-RE |  | GTGGCAGAGTAAGGGATGGA  GGGGATGAACCATTGACAAC  CACCCCAAAAGTTCAGTCGT  TGTTTAGCTCCGCCAACTCT  AAAGCTATTTTGCCCAAGCA  TTCAGCACTGCTCCTTGAAA  CAATTTAAGAAGGCGCTGGT  CTGGTCTACCCCAAGTGCAT |  |
| VCAN-AS1-FW  VCAN-AS1-RE |  | TCCTCCATTCAGGCCTTTCTTG  GCGACTTTCAAACCATCAAGTG |  |
| FENDRR-FW  FENDRR-RE |  | GCGCACAGACCCAGGATTT  CACGGGCAGAGCTGGTTT |  |
| lncNFAT5-1-FW  lncNFAT5-1-RE |  | TGCAGATGTACGGTGTGGAT  AGTGCAGTGGTGTGATCTCG |  |
| TBX2-AS1:1-FW  TBX2-AS1:1-RE |  | GATCTGGTGGAGGGAACTGA  TAATGGAAAAGCCGTGGAAA |  |
| lnc-DLD-1-FW  lnc-DLD-1-RE |  | CCCAGAGGTGTGTGTTTGTG  AGTCCGCAGTTCATGCTCTT |  |
| MRGPRF-AS1-FW  MRGPRF-AS1-RE |  | GAGCTACCTGCAGCTGTCCT  TTCTGCTTCCAAAGCCATCT |  |
| RNU2-FW  RNU2-RE |  | CATCGCTTCTCGGCCTTTTG  TGGAGGTACTGCAATACCAGG |  |
| GAPDH-FW  GAPDH-RE |  | GCACCGTCAAGGCTGAGAAC  TGGTGAAGACGCCAGTGGA |  |
| β-actin-FW |  | GCCGGGACCTGACTGACTAC |  |
| β-actin-RE |  | TTCTCCTTAATGTCACGCACGAT |  |
| FW: Forward, RE: Reverse  Species: Human  MIR100HG:29, VCAN-AS1:6, TBX2-AS1:1,MRGPRF-AS1:2, and FENDRR variant 2 transcripts were used for primer design. | | |  |

**Supplementary Table 2: Numbers of de-regulated mRNAs by hypoxia and/or TGFβ1 treatment based on their fold changes**

| **Fold Change** | **Hypoxia** | | **TGFβ1** | | **Hypoxia + TGFβ1** | |
| --- | --- | --- | --- | --- | --- | --- |
|  | **Up** | **Down** | **Up** | **Down** | **Up** | **Down** |
| **2>10** | 410 | 359 | 357 | 453 | 1,171 | 1,265 |
| **10>50** | 6 | 9 | 21 | 15 | 111 | 96 |
| **50>100** | 0 | 1 | 5 | 2 | 4 | 5 |
| **>100** | 0 | 0 | 3 | 2 | 18 | 6 |
| **Total** | **416** | **369** | **386** | **472** | **1,304** | **1,372** |
| **Cumulative total** | **785** | | **858** | | **2676** | |

**Supplementary Table 3: Myofibrobalst marker expression in HPFs exposed to hypoxia and/or TGFβ1 from RNA sequencing data**

| **Gene** | **FPMK** | | | | **Fold Change** | | |
| --- | --- | --- | --- | --- | --- | --- | --- |
|  | **Control** | **TGFβ1** | **Hypoxia** | **Hypoxia + TGFβ1** | **TGFβ1** | **Hypoxia** | **Hypoxia + TGFβ1** |
| **ACTA2 (α-SMA)** | 76 | 138****^,####^ | 83 | 490****^, ####, $$$$^ | 1.8 | 1.1 | 6.4 |
| **COL1A1** | 775 | 3,698** | 1648* | HIDATA^b^ | 4.8 | 2.1 | HIDATA^a^ |
| **COL3A1** | 370 | 805** | 446 | 644**^, #^ | 2.2 | 1.2 | 1.7 |
| **FN1** | 1,766 | HIDATA^b^ | 2051 | HIDATA^b^ | HIDATA^b^ | 1.2 | HIDATA^a^ |
| **CTGF** | 14 | 160****^,####^ | 41**** | 692****^,####, $$$$^ | 11.0 | 2.8 | 47.4 |

^a^HIDATA: Genes with a large number of aligned reads (more than 20 ×10^6^). Which were omitted from the expression analysis. These genes have extremely high expression.

*P<0.05, **P<0.01, ****P<0.0001 vs. Control. ^#^P<0.05, ^####^P<0.0001 vs. Hypoxia, ^$$$$^P<0.0001 vs. TGFβ1. P value were obtained from Cuffdiff analysis of RNA seq.

FPKM= Fragments Per Kilobase of transcripts per Million mapped reads

**Supplementary Table 4: Genes in HIF signaling pathway up-regulated by TGFβ1**

| **Gene Abbreviation** | **Gene Name^a^** | **Function^a^** | **Fold change**  **(RNA_seq)** |
| --- | --- | --- | --- |
| SLC2A1 | Solute Carrier Family 2 Member 1 | Encodes a major glucose transporter in the mammalian blood-brain barrier | 2.45 ± 0.03 |
| SERPINE1 | Serpin Family E Member 1 | Encodes a member of the serine proteinase inhibitor (serpin) superfamily, main inhibitor of tissue plasminogen activator and urokinase | 5.95 ± 0.14 |
| PFKL | Phosphofructokinase, Liver Type | Codes the liver (L) subunit of an enzyme that catalyzes D-fructose 6-phosphate to D-fructose 1,6-bisphosphate conversion in glycolysis | 2.24 ± 0.12 |
| IGF1 | Insulin Like Growth Factor 1 | Function is similar to insulin, has a growth activity | 7.36 ± 1.28 |
| EIF4EBP1 | Eukaryotic Translation Initiation Factor 4E Binding Protein 1 | Encodes one member of a family of translation repressor proteins, repress the translation | 2.03 ± 0.17 |
| EDN1 | Endothelin 1 | Encodes a preproprotein, which is a vasoconstrictor | 3.29 ± 0.60 |
| ANGPT1 | Angiopoietin 1 | Encodes a secreted glycoprotein in angiopoietin family, which is involved in vascular development and angiogenesis | 2.07 ± 0.09 |

^a^Source: http://www.genecards.org/

| **Gene Abbr.** | **Gene Name^a^** | **Function^a^** | **Fold change (RNA seq)** |
| --- | --- | --- | --- |
| VEGFA | Vascular Endothelial Growth Factor A | A member of the PDGF/VEGF growth factor family and encodes a heparin-binding protein, induces endothelial cell proliferation and migration | 5.89 ± 0.03 |
| SLC2A1 | Solute Carrier Family 2 Member 1 | Encoded protein is a major glucose transporter in the mammalian blood-brain barrier | 2.56 ± 0.03 |
| TIMP1 | Tissue Inhibitor Of Metalloproteinases 1 | Natural inhibitors of the matrix metalloproteinases | 4.08 ± 0.08 |
| SERPINE1 | Serpin Family E Member 1 | Encodes a protein in serine proteinase inhibitor (serpin) superfamily. Inhibits fibrinolysis by inhibiting plasminogen activator and urokinase | 16.39 ± 0.14 |
| TF | Transferrin | Encodes glycoprotein which binds with ferric iron | 2.26 ± 0.03 |
| IL6 | Interleukin 6 | Encodes IL6 cytokine, which acts in inflammation in body | 10.93 ± 0.07 |
| IGF1 | Insulin Like Growth Factor 1 | Function is similar to insulin, has a growth activity | 12.42 ± 1.28 |
| MKNK2 | MAP Kinase Interacting Serine/Threonine Kinase 2 | Encodes a member of the calcium/calmodulin-dependent protein kinases (CAMK) Ser/Thr protein kinase family, mainly involved in phosphorylation of eukaryotic initiation factor 4G (elF4G) | 3.05 ± 0.01 |
| EIF4EBP1 | Eukaryotic Translation Initiation Factor 4E Binding Protein 1 | Encodes one member of a family of translation repressor proteins, repress the translation | 9.83 ± 0.17 |
| HKDC1 | Hexokinase Domain Containing 1 | Encoded a protein involved in insulin regulation of translation and carbon metabolism in cancer | 4.13 ± 0.07 |
| PDK1 | Pyruvate Dehydrogenase Kinase 1 | Encodes a mitochondrial enzyme, which catalyzes the pyruvate decarboxylation | 2.17 ± 0.18 |
| AKT3 | AKT Serine/Threonine Kinase 3 | Regulates cell signaling in response to insulin and growth factors | 2.39 ± 0.06 |
| HK2 | Hexokinase 2 | Phosphorylates glucose to glucose 6-phosphate | 2.36 ± 0.03 |
| IGF1R | Insulin Like Growth Factor 1 Receptor | Binds to insulin with high affinity | 2.80 ± 0.1 |
| EDN1 | Endothelin 1 | Encodes a preproprotein, which is a vasoconstrictor | 6.53 ± 0.6 |
| PRKCA | Protein Kinase C Alpha | Belongs to the family of serine-and threonine specific protein kinases, phosphorylates many proteins | 3.01 ± 0.02 |

^a^Source: http://www.genecards.org/

**Supplementary Table 5: Genes in HIF signaling pathway up-regulated by hypoxia +TGFβ1**

**Supplementary Table 6: Genes in TGFβ signaling pathway up-regulated by hypoxia**

| **Gene Abbreviation** | **Gene Name^a^** | **Function^a^** | **Fold Change**  **(RNA_seq)** |
| --- | --- | --- | --- |
| BMP4 | Bone morphogenic protein 4 | Encodes a secreted ligand of proteins of TGFβ superfamily | 2.82 ± 0.04 |
| INHBE | Inhibin Beta E subunit | Encodes a member of proteins of TGFβ superfamily | 2.19 ± 0.35 |
| GDF5 | Growth Differentiation Factor 5 | Encodes a secreted ligand of proteins of TGFβ superfamily | 3.72 ± 0.22 |
| ID4 | Inhibitor of DNA binding 4 | Encodes a member of the inhibitor of DNA binding protein family. | 3.40 ± 0.64 |
| BMP5 | Bone morphogenic protein 5 | Encodes a secreted ligand of proteins of TGFβ superfamily | 4.85 ± 1.07 |
| THBS1 | Thombrospondin 1 | An adhesive glycoprotein, mediates cell-cell and cell-matrix interactions, binds with fibrinogen, fibronectin, laminin, type V collagen and integrin alpha-V/beta-I | 2.79 ± 0.07 |

^a^Source: <http://www.genecards.org/>

| **Gene Abbreviation** | **Gene Name^a^** | **Function^a^** | **Fold Change**  **(RNA seq)** |
| --- | --- | --- | --- |
| INHBA | Inhibin Beta A Subunit | Encodes a member of proteins of TGFβ superfamily | 2.65 ± 0.06 |
| INHBE | Inhibin Beta E subunit | Encodes a member of proteins of TGFβ superfamily | 202.93 ± 0.64 |
| FST | Follistatin | Inhibits follicle-stimulating hormone release | 3.86 ± 0.04 |
| SMAD7 | SMAD Family Member 7 | Binds with SMURF2 and initiates the degradation of TGFβ receptor type 1 | 2.34 ± 0.12 |
| MYC | V-Myc Avian Myelocytomatosis Viral Oncogene Homolog | Encodes a protein with multifunction, involved in cell cycle progression, apoptosis and cellular transformation | 4.55 ± 0.05 |
| TGFB1 | Transforming Growth Factor Beta 1 | Binds with TGF-beta receptor which initiates SMAD-based gene transcription | 3.38 ± 0.09 |
| CDKN2B | Cyclin Dependent Kinase Inhibitor 2B | Binds with CDK4 and CDK6, prevents CDK kinases activation and cell cycle progression | 3.36 ± 0.06 |
| ID4 | Inhibitor Of DNA Binding 4, HLH Protein | Acts as a tumor suppressor | 7.59 ± 0.35 |
| PITX2 | Paired Like Homeodomain 2 | Acts as a transcription factor, regulates the gene expression of procollagen lysyl hydroxylase | 4.76 ± 0.27 |
| E2F5 | E2F Transcription Factor 5 | Belongs to E2F transcription factors family, acts as a transcriptional activator. | 4.03 ± 0.30 |
| THBS1 | Thombrospondin 1 | An adhesive glycoprotein, mediates cell-cell and cell-matrix interactions, binds with fibrinogen, fibronectin, laminin, type V collagen and integrin alpha-V/beta-I | 4.18 ± 0.05 |
| SMURF2 | SMAD Specific E3 Ubiquitin Protein Ligase 2 | Involved in ubiquitin-dependent protein degradation | 2.63 ± 0.05 |

**Supplementary Table 7: Genes in TGFβ signaling pathway up-regulated by hypoxia+TGFβ1**

^a^Source: http://www.genecards.org/

**Supplementary Table 8: The number of lncRNAs altered by hypoxia and/or TGFβ1 treatments based on their fold changes.**

| **Fold Change** | **Hypoxia** | | **TGFβ1** | | **Hypoxia + TGFβ1** | |
| --- | --- | --- | --- | --- | --- | --- |
|  | **Up** | **Down** | **Up** | **Down** | **Up** | **Down** |
| **2>10** | 99 | 110 | 76 | 63 | 337 | 237 |
| **10>50** | 3 | 4 | 2 | 2 | 45 | 23 |
| **50>100** | 1 | 3 | 1 | 2 | 2 | 4 |
| **>100** | 2 | 0 | 4 | 0 | 11 | 10 |
| **Total** | **105** | **117** | **83** | **67** | **395** | **274** |
| **Cumulative Total** | **222** | | **150** | | **669** | |

**Supplementary Table 9: Up-regulated and down-regulated lncRNAs in hypoxia + TGFβ1 treatment based on selection criteria**

**Supplementary Table 9. Up-regulated and down-regulated lncRNAs in hypoxia + TGFβ treatment based on selection criteria**

|  | **Gene^a^** | **Transcripts^a^** | **Transcript Size^a^** | **locus** | **FPKM** | | | | **Fold Change** | | | **Lung Expression (NONCODE)^b^** |
| --- | --- | --- | --- | --- | --- | --- | --- | --- | --- | --- | --- | --- |
|  |  |  |  |  | **Control** | **Hypoxia** | **TGFβ1** | **Hypoxia +TGFβ1** | **Fold Change_ Hypoxia** | **Fold Change_ TGFβ1** | **Fold Change_**  **Hypoxia+TGFβ1** |  |
| **Up** | MIR100HG | 14 | 387-3370 bp^c^ | chr11:121899062-121987031 | 1,431 | 1,893* | 2,036** | 7,345****^,####,$$$$^ | 1.32 | 1.42 | 5.13 | 0-28.53 |
|  | DDIT4-AS1 | 1 | 847 bp | chr10:74034672-74035738 | 661 | 940**** | 657^####^ | 4,058****^,####,$$$$^ | 1.42 | 0.99 | 6.14 | 105.87 |
|  | lnc-RPS27L-1 | 1 | 691 bp | chr15:63362223-63364110 | 637 | 781*** | 1904****^,####^ | 9,471****^,####,$$$$^ | 1.23 | 2.99 | 14.86 | 46.27 |
|  | lnc-DKK3-1 | 1 | 500 bp | chr11:12282972-12284720 | 332 | 661**** | 7041**** | 4,947****^,####,$$$$^ | 1.99 | 2.12 | 14.90 | 21.75 |
|  | VCAN-AS1 | 2 | 424-453 bp^c^ | chr5:82827170-82877139 | 282 | 479*** | 2593****^,####^ | 4,338****^,####,$$$$^ | 1.70 | 9.19 | 15.36 | 0-45.26 |
| **Down** | FENDRR | 15 | 355-3379 bp^c^ | chr16:86508134-86542705 | 1,686 | 950**** | 1,029*** | 275****^,####,$$$$^ | 1.78 | 1.64 | 6.13 | 0.43-2.83 |
|  | lnc-NFAT5-1 | 1 | 575 bp | chr16:69743776-69744486 | 1,540 | 1,197*** | 427****^,####^ | 241****^,####,$$$$^ | 1.29 | 3.60 | 6.39 | 9.4 |
|  | TBX2-AS1 | 6 | 330-1184 bp^c^ | chr17:59439028-59488916 | 1,144 | 873 | 580*** | 220****^,####,$$$$^ | 1.31 | 1.97 | 5.20 | 0-10.89 |
|  | lnc-DLD-1 | 1 | 625 bp | chr7:107582560-107583185 | 336 | 192**** | 269*^,###^ | 43****^,####,$$$$^ | 1.75 | 1.25 | 7.74 | 1.55 |
|  | MRGPRF-AS1 | 3 | 542-1568 bp^c^ | chr11:68779750-68785915 | 139 | 46**** | 99*^,##^ | 19****^,##,$$$$^ | 3.05 | 1.41 | 7.14 | 0-1.10 |

^a^LNCpedia database(<https://lncipedia.org/>) version 4.0 was used for gene names, transcripts and transcript sizes.

^b^NONCODE database (<http://www.noncode.org/>) NONCODE2016 version was used for lung expression.

^c^These transcripts have multiple transcripts and their lowest and highest transcript sizes were given in the table.

*P<0.05, **P<0.01,***P<0.001 ****P<0.0001 vs. Control. ^##^P<0.01^,###^P<0.001, ^####^P<0.0001 vs. Hypoxia, ^$$$$^P<0.0001 vs. TGFβ1. P value were obtained from Cuffdiff analysis of RNA seq.
